# Opioid therapy duration before naldemedine treatment is a significant independent risk of diarrhea: a retrospective cohort study

**DOI:** 10.1186/s40780-020-00187-3

**Published:** 2021-02-01

**Authors:** Akiharu Okamoto, Kenji Ikemura, Eri Mizutani, Takuya Iwamoto, Masahiro Okuda

**Affiliations:** 1 Department of Pharmacy, Mie University Hospital, 2-174 Edobashi, Tsu, Mie 514-8507 Japan; 2 Department of Clinical Pharmaceutics, Mie University Graduate School of Medicine, 2-174 Edobashi, Tsu, Mie 514-8507 Japan; 3 Department of Pharmacy, Osaka University Hospital, 2-2 Yamadaoka, Suita, Osaka 565-0871 Japan

**Keywords:** Naldemedine, Diarrhea, Opioid-induced constipation, Peripherally acting μ-opioid receptor antagonist

## Abstract

**Background:**

The most common adverse event (AE) associated with opioid analgesics is opioid-induced constipation (OIC). Naldemedine (NAL) is widely used for the treatment of OIC. However, diarrhea has been reported as the most common treatment-emergent AE of NAL, and little is known about the risk factors associated with the development of diarrhea during NAL administration. This study examined the risk factors for NAL-induced diarrhea via a retrospective chart review of hospitalized patients.

**Methods:**

The data of 101 hospitalized adult patients who received NAL for the first time for the treatment of OIC at Mie University Hospital between June 2017 and December 2018 were extracted from electronic medical records. According to the inclusion and exclusion criteria, 70 of the 101 patients were enrolled in this study. Diarrhea was defined as “diarrhea” on the medical record within 2 weeks of NAL administration. Univariate and multivariate analyses were performed to identify risk factors for the development of diarrhea in patients receiving NAL.

**Results:**

Twenty-two of the 70 patients enrolled (31%) developed diarrhea within 2 weeks of NAL administration. The median duration (range) of NAL treatment before diarrhea onset was 3 (1–12) days. Patients with diarrhea had a significantly longer duration of opioid therapy before NAL administration than patients without diarrhea (*P*=0.002). Multivariate logistic regression analysis indicated that the independent risk factors for the development of NAL-induced diarrhea were NAL administration after more than 17 days of opioid therapy (odds ratio [OR]=7.539; *P*=0.016) and pancreatic cancer (OR=6.217; *P*=0.025). In fact, the incidence of diarrhea in patients who were administered NAL within a day of opioid therapy was significantly lower than that in patients who were administered NAL after more than 17 days of opioid therapy (13% vs. 54%, *P*=0.030).

**Conclusions:**

These results suggested that a prolonged duration of opioid therapy prior to NAL initiation is associated with increased incidence of diarrhea.

**Supplementary Information:**

The online version contains supplementary material available at 10.1186/s40780-020-00187-3.

## Background

The most common adverse event (AE) associated with opioid analgesics include opioid-induced constipation (OIC). The incidence of OIC has been reported in approximately 40–90% of patients treated with the opioids for pain for over a long period [1, 2]. Generally, the administration of laxatives, fiber intake, and exercise therapy are first applied to OIC, however, they do not have sufficient effects in many cases [3–5].

Naldemedine (NAL) is a peripherally acting μ-opioid receptor antagonist (PAMORA) that is widely used for OIC treatment [6–10]. PAMORAs are effective in patients with laxative-resistant OIC and their large side chains helps them attain bulky molecular sizes. Therefore, they can avoid the infiltration of the blood-brain barrier at therapeutic doses, resulting in the maintenance of the central analgesic effects of opioids [11].

In a phase III randomized placebo-controlled trial of NAL, the frequency of spontaneous bowel movements per week in patients receiving NAL was significantly higher compared with that in patients receiving placebo [6]. However, gastrointestinal disorders are the most common treatment-emergent AEs of NAL. The AE that was most commonly associated with NAL use was diarrhea (20%), and NAL administration was discontinued in 5% of patients with diarrhea [6]. Moreover, the meta-analysis demonstrated that NAL and lubiprostone were associated with an increased risk of AE in comparison with other laxatives [8]. Thus, the prediction of diarrhea associated with NAL could contribute to the effective use of NAL with less treatment discontinuation. However, little is known about the risk factors of NAL-induced diarrhea.

The US package insert (Symproic®) mentioned that patients administered opioids for less than 4 weeks may be less responsive to NAL based on two randomized double-blind placebo-controlled trials. In these trials, non-cancer patients received stable opioids for at least 4 weeks [12]. Other phase IIb and phase III randomized placebo-controlled trials of NAL, including cancer patients with OIC, also restricted enrolled patients to opioids for more than 2 weeks [6, 13]. These evidences indicate that there is room for consideration in the efficacy and safety of NAL in patients who had a short duration of opioid therapy before NAL administration.

In the present study, the risk factors for NAL-associated diarrhea were examined via a retrospective chart review of the hospitalized patients who received NAL without restriction of long-term opioid use.

## Methods

### Patients selection

This retrospective study included hospitalized patients (> 20 years old) who received NAL (Symproic® tablets 0.2 mg, SHIONOGI & CO., LTD.) for the first time for the treatment of OIC in Mie University Hospital between June 2017 and December 2018. Patients were excluded if they had missing data, colostomy, stool extraction, and NAL dosing period of less than 3 days.

### Evaluation of diarrhea

Diarrhea was defined as ‘diarrhea’ on the medical record within 2 weeks after initiation of NAL administration. In a previous randomized controlled trial, this threshold was referred to as the survey period of diarrhea [6]. The severity of diarrhea was evaluated according to the Common Terminology Criteria for Adverse Events version 4.0 (CTCAE). Patients’ data were excluded from the evaluation of severity of diarrhea if patients had no data regarding the frequency of stools within 3 days before the start of NAL administration (*n*=5).

### Data collection for eligible patients

Patient characteristics were extracted from clinical records: age, sex, body weight, Eastern Cooperative Oncology Group performance status (PS), creatinine clearance calculated using the Cockcroft-Gault equation, NAL dose, use of opioid analgesics, opioid dose, duration of opioid therapy before NAL administration, and co-administrated drugs (risk drugs for constipation, proton-pump inhibitor (PPI) or H_2_-blocker, laxative, anticancer drug, antimicrobial agent), and tubal feeding during NAL administration. The value of serum creatinine < 0.6 mg/dL was substituted for serum creatinine = 0.6 mg/dL [14]. The drugs listed as the cause of constipation in the Clinical Guidelines for Gastrointestinal Symptoms in Cancer Patients, which was edited by the Japanese Society for Palliative Medicine, were defined as the risk drugs for constipation: scopolamine hydrobromide, phenothiazine antipsychotics, tricyclic antidepressants, anti-Parkinson’s drugs, antacids, diuretics, anticonvulsants, iron, antihypertensive drugs, and anticancer drugs. Morphine-equivalent doses were calculated using established methods (oral morphine 30 mg = intravenous (IV) morphine 15 mg = oral oxycodone 20 mg = IV oxycodone 15 mg = fentanyl 0.3 mg = oral hydromorphone 6 mg = oral tramadol 150 mg and oral methadone 45 mg = oral morphine 450 mg) in ethical drug package insert established by the Pharmaceuticals and Medical Devices Agency and the Clinical Guidelines for Gastrointestinal Symptoms in Cancer Patients which was edited by the Japanese Society for Palliative Medicine.

### Statistical analyses

Statistical comparisons between two groups in the presence and absence of diarrhea were performed using the Mann-Whitney U test and Fisher’s exact test for continuous and categorical variables, respectively. The multivariate logistic regression model was adjusted for the following potentially confounding factors for the development of diarrhea, which were detected as *P* < 0.20 in the univariate analyses (female, PS, opioid dose, NAL administration after more than 17 days of opioid therapy, co-administration of PPI or H_2_-blocker, fentanyl, and pancreatic cancer), using the forced entry method. Statistical analyses were performed using IBM SPSS statistics version 26.0 (Armonk, NY, USA). Significance was established at a *P* value < 0.05. Cutoff values of duration of opioid therapy before NAL administration were determined by the receiver operating characteristics (ROC) curve method with JMP® version 12.0.1 (SAS Institute Inc., Cary, NC, USA).

## Results

### Patients’ characteristics

**Fig. 1:**
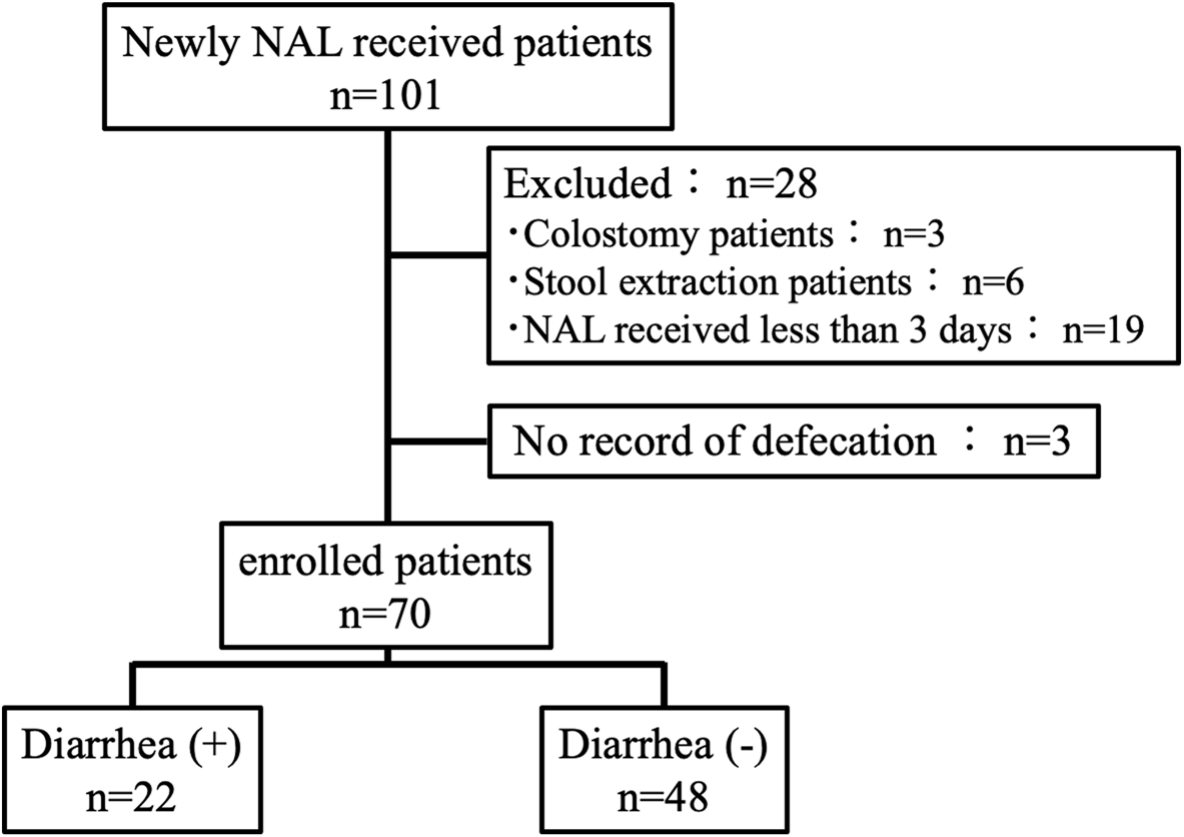
Flow chart of patient selection

The flow chart of patient selection is shown in Fig. 1. Total of 101 hospitalized patients were screened, and 31 patients who had missing data (*n*=3), colostomy (*n*=3), stool extraction (*n*=6), and NAL dosing period of less than 3 days (*n*=19) were excluded from the study patients. Nineteen patients who had NAL dosing period of less than 3 days were excluded for the following reasons: hospital stay of less than 2 days (10 cases), discontinuation of opioid (3 cases), defecation without oral administration (3 cases), discontinuation due to peristaltic pain after NAL administration (1 case), use only for one time (1 case), and oral administration not possible (1 case). After considering the inclusion and exclusion criteria, 70 patients were enrolled in the present study. Patient characteristics are summarized in Table 1. The median age of the patients was 67 (range, 30–85) years, and 30 patients (43%) were female. The duration of opioid therapy before NAL administration (day [range]) in patients with diarrhea was significantly longer than that in patients without diarrhea (27 [0–1365] vs. 4 [0–268], *P*=0.002). In contrast, there were no significant differences in other characteristics between patients with and without diarrhea. In 60 eligible patients, excluding the patients who could not be evaluated bowel movements before NAL administration (*n*=10), significant differences regarding bowel movements during 7 days before NAL administration were not observed between the patients with and without diarrhea (*n*=16 [89%] vs *n*=35 [83%], *P*=0.710).

**Table 1:** Patient Characteristics

Characteristics	All patients (*n*=70)	Diarrhea (+) (*n*=22)	Diarrhea (−) (*n*=48)	*P* value
Age (years)	67 [30–85]	63 [37–81]	67 [30–85]	0.714
Female	30 (43)	13 (59)	17 (36)	0.074
Body weight (kg)	52.5 [31.0–76.8]	51.3 [38.6–72.6]	54.6 [31.0–76.8]	0.786
CCr (mL/min)	67.5 [5.1–140.0]	71.5 [27.7–120.5]	63.0 [5.1–140.0]	0.305
Performance status (3–4)	12 (17)	6 (27)	6 (13)	0.174
NAL dose (μg/kg)	3.8 [2.6–6.5]	3.9 [2.8–5.2]	3.7 [2.6–6.5]	0.849
Opioid dose (morphine base, mg)	17.5 [2.5–390.0]	30.0 [7.5–390.0]	15.0 [2.5–180.0]	0.094
Duration of opioid therapy before NAL administration (day)	6 [0–1365]	27 [0–1365]	4 [0–268]	0.002
Co-administrated drugs				
Drugs with risk of constipation	61 (87)	19 (86)	42 (88)	1.000
PPI or H_2_-blocker	49 (70)	18 (82)	31 (65)	0.171
Laxative	58 (83)	20 (91)	38 (79)	0.316
Oral anticancer drug	13 (19)	6 (27)	7 (15)	0.320
Intravenous anticancer drug	30 (43)	10 (45)	20 (42)	0.800
Antimicrobial agent	36 (51)	12 (55)	24 (50)	0.800
Tubal feeding	5 (7)	2 (9)	3 (6)	0.646
Opioid analgesics				
Oxycodone	49 (70)	16 (73)	33 (69)	0.787
Morphine	3 (4)	0 (0)	3 (6)	0.547
Fentanyl	7 (10)	4 (18)	3 (6)	0.195
Tramadol	9 (13)	1 (5)	8 (17)	0.255
Methadone	1 (1)	1 (5)	0 (0)	0.314
Hydromorphone	1 (1)	0 (0)	1 (2)	1.000
Primary tumor				
Pancreatic	12 (17)	6 (27)	6 (13)	0.174
Head and neck	8 (11)	2 (9)	6 (13)	1.000
Lung	6 (9)	2 (9)	4 (8)	1.000
Breast	5 (7)	1 (5)	4 (8)	1.000
Soft tissue	5 (7)	0 (0)	5 (10)	0.173
Hepatic	4 (6)	2 (9)	2 (4)	0.585
Stomach	3 (4)	1 (5)	2 (4)	1.000
Colorectal	2 (3)	0 (0)	2 (4)	1.000
Others and non-cancer	25 (36)	8 (36)	17 (35)	1.000

Values are presented as median [range] or number (%)

*CCr* Creatinine clearance, *NAL* Naldemedine, *PPI* Proton-pump inhibitor

### Occurrence rate and severity of diarrhea

The number of patients developing diarrhea, the duration of NAL treatment before diarrhea and severity of diarrhea are summarized in Table 2. Within 2 weeks after the start of NAL administration, 22 patients (31%) developed diarrhea. The median duration of NAL administration before diarrhea [range] was 3 [1–12] days. Additionally, patients who had no data regarding the frequency of stools within 3 days before NAL administration were excluded (*n*=5), and we evaluated the severity of diarrhea in 17 patients receiving NAL. Grades 1 and 2 diarrheas were observed in 13 patients (76%) and 4 patients (24%), respectively, while grades 3 and 4 were not observed.

**Table 2:** Incidence of diarrhea, duration of naldemedine treatment before diarrhea and severity of diarrhea in patients receiving NAL

	Number (%) or median [range]
Incidence of diarrhea (%)	22 (31%)
Duration of naldemedine treatment before diarrhea (day)	3 [1–12]
CTCAE Grade (*n*=17)	
1	14 (76%)
2	3 (24%)
3	0 (0%)
4	0 (0%)

*CTCAE* Common Terminology Criteria for Adverse Events

### Risk factors for the development of diarrhea

Although we investigated correlation between variables (female, PS, opioid dose, NAL administration after more than 17 days of opioid therapy, co-administration of PPI or H_**2**_-blocker, fentanyl, and pancreatic cancer), strong correlations were not observed (correlation coefficient: | r | ≤ 0.524). Multivariate logistic regression analysis was conducted to investigate the risk factors for the development of diarrhea in patients receiving NAL (Table 3). The cutoff value (area under the ROC curve, AUC) for the duration of opioid therapy before NAL administration was 17 days (0.73). The sensitivity and specificity were 64 and 77%, respectively. As shown in Table 3, multivariate analysis revealed that the independent risk factors for diarrhea were NAL administration after more than 17 days of opioid therapy (odds ratio [OR]=7.539; *P*=0.016) and pancreatic cancer (OR=6.217; *P*=0.025). Moreover, these significant variables were also detected as independent risk factors in the multivariate analyses using forward and backward stepwise selection methods.

**Table 3:** Multivariate logistic regression analysis for risk factors of diarrhea in patients receiving naldemedine

Variables	Odds ratio	95% CI	*P* value
Female	3.012	0.887–10.228	0.077
Performance status (3–4)	2.806	0.567–13.887	0.206
Opioid dose (morphine base, mg)	1.001	0.987–1.016	0.853
Naldemedine administration after more than 17 days of opioid therapy	7.539	1.446–39.305	0.016
PPI or H_2_-blocker	2.323	0.476–11.334	0.297
Fentanyl	0.552	0.082–3.720	0.541
Pancreatic cancer	6.217	1.257–30.378	0.025

*CI* Confidence interval, *PPI* Proton-pump inhibitor

### Relationship between the incidence of diarrhea and duration of opioid therapy before NAL administration

**Fig. 2:**
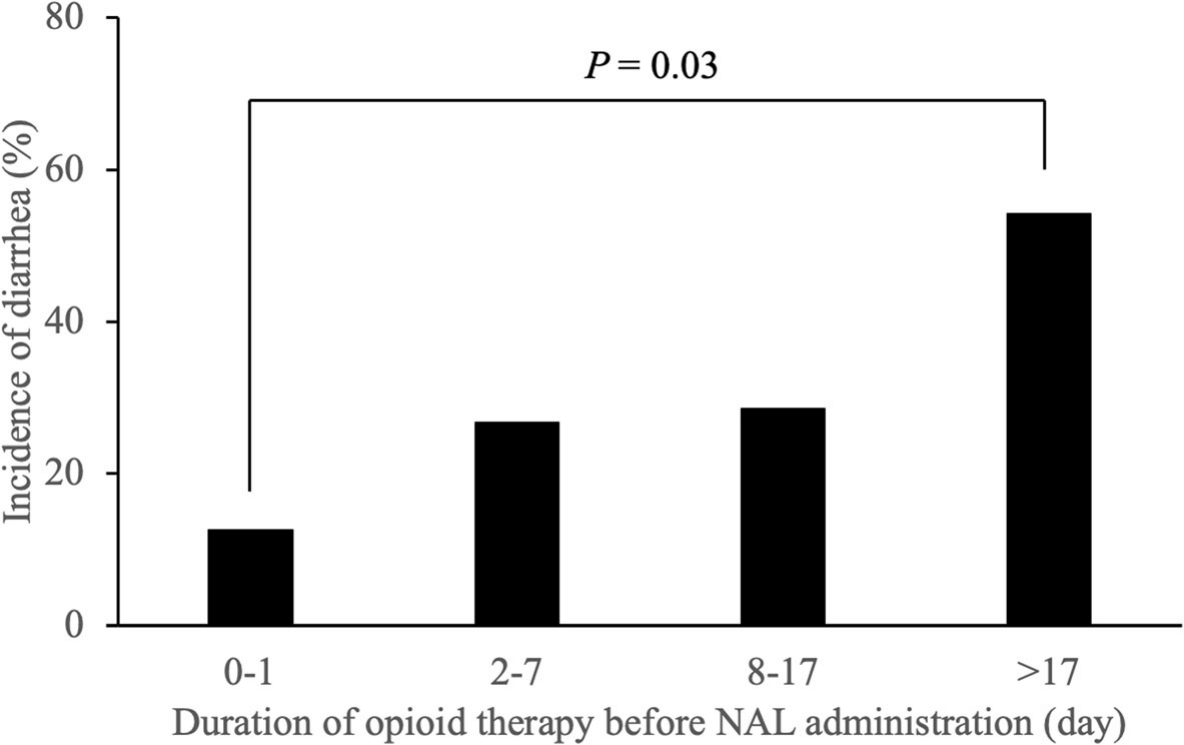
Relationship between the duration of opioid therapy before naldemedine administration and the incidence of diarrhea

The relationship between the incidence of diarrhea and duration of opioid therapy before NAL administration is shown in Fig. 2. The incidence of diarrhea in patients who started receiving NAL within 0–1, 2–7, 8–17, and > 17 days of opioid therapy was 13, 27, 29, and 54%, respectively. The incidence of diarrhea in patients who started receiving NAL within 1 day of opioid therapy was significantly lower than that in patients who started after more than 17 days (*P*=0.030). Prolonged opioid therapy before NAL administration tended to increase the incidence of diarrhea.

## Discussion

Little is known about the effect of the duration of opioid therapy on NAL-induced diarrhea. This study demonstrated that a prolonged duration of opioid therapy before NAL administration increased the risk of diarrhea in patients who received NAL. Moreover, multivariate analysis indicated that NAL administration after more than 17 days of opioid therapy and pancreatic cancer were significant independent risks factors for NAL-induced diarrhea.

In previous randomized controlled trials, patients who started NAL treatment after 2 weeks or more of opioid therapy were enrolled [6, 13]. In the present study, the duration of opioid therapy before NAL administration in enrolled patients was 0 to 1365 days (Table 1), and 45 patients underwent opioid therapy for less than 2 weeks before NAL administration. In contrast, one of the aforementioned previous randomized controlled study focused on a patient population with lung (43%), breast (23%), colorectal (3%), and other cancers (31%) [6]. As shown in Table 1, we analyzed patients with various cancer types, including pancreatic cancer (17%), head and neck cancer (11%), lung cancer (9%), breast cancer (7%), and soft tissue tumor (7%). Owing to differences in the duration of opioid therapy before NAL administration and in cancer types between our study and the above previous randomized control trials, it was conceived that we could evaluate the unexamined risk factors for diarrhea associated with NAL in the present study.

As shown in Fig. 2, the incidence of diarrhea in patients starting NAL administration within a day of opioid therapy was significantly lower than in patients starting it after more than 17 days (13% vs. 54%, *P*=0.03). A previous study reported that the incidences of diarrhea in patients who started NAL treatment within 3 days and more than 3 days of opioid therapy were 4 and 22%, respectively [15]. Another study reported that the incidence of diarrhea (40%) significantly increased in patients who started NAL treatment after more than 7 days of opioid therapy [16]. As our results and previous study results indicate that prolonged duration of opioid therapy before NAL administration affects the incidence of NAL-induced diarrhea. On the other hand, there is a difference in the cutoff value of duration of opioid therapy before NAL administration between studies. In previous studies [15, 16], the cutoff values for duration of opioid therapy before NAL administration were not clearly mentioned. By contrast, we statistically calculated the cutoff value by ROC curve method. Therefore, our estimated cutoff value should be more reliable than those in previous studies.

As shown in Table 3, multivariate analysis revealed that pancreatic cancer is also a risk factor for diarrhea in patients receiving NAL. Diarrhea often develops due to pathological conditions, surgical complications and/or decreased secretion of pancreatic enzymes, in patients with pancreatic cancer [17, 18]. As shown in Supplementary Table 1, the number of pancreatic cancer patients who received chemotherapy with and without diarrhea were 4 cases (Gemcitabine (GEM) plus S^− 1^ [2cases], S-1 [2cases]) and 3 cases (GEM, nanoparticle albumin-bound paclitaxel plus GEM, FOLFIRINOX), respectively. In previous studies, the incidence of diarrhea in S-1 plus GEM is significantly higher than that in GEM alone [19] and Todaka et al. [20] reported that S-1 administration is reported to be a risk of diarrhea. The incidence of diarrhea in FOLFIRINOX is also reported to be significantly higher than that in GEM alone [21]. However, in our study, only one patient underwent FOLFIRINOX regimen, and the patient did not develop diarrhea during the study period. Additionally, the proportion of pancreatic cancer patients in our study was higher than that in the previous study, and as a result, the incidence of diarrhea in our study (31%) was higher than that in the previous report (20%) [6]. However, the number of eligible patients was small to prove the involvement of NAL administration in the development of diarrhea in the patients with pancreatic cancer. Therefore, further study will be needed to reveal the implication of pancreatic cancer on the development of NAL-induced diarrhea.

Opioid dose (morphine base, mg [range]) in patients more than 17 days of opioid therapy was significantly higher than in patients who started receiving NAL ≤17 days of opioid therapy (52.5 [15–390] vs 15 [2.5–45], *P*< 0.001). Although it is considered that opioid dose increases as opioid therapy prolonged, opioid dose was not extracted as an independent risk factor for NAL-induced diarrhea in the multivariate analysis (Table 3). Therefore, the opioid dose could not significantly affect the development of diarrhea after NAL administration.

Laxatives and tubal feedings may affect the incidence of diarrhea depending on their initiation timing. In the present study, there were no significant differences in the number of patients who started laxatives after NAL administration between the patients with and without diarrhea (3 [13%] vs 5 [10%], *P*=0.700). Only one patient had started tubal feeding during the study period, and diarrhea was not observed. Thus, we speculated that these factors could have little influence on the development of diarrhea.

Diarrhea is an opioid withdrawal syndrome occurring after sudden reduction or discontinuation of opioids [22]. The severity and duration of withdrawal syndromes widely differ according to the type, dose, and duration of opioid administered [23]. A previous study reported that the degree of withdrawal symptoms such as diarrhea by an opioid antagonist, naloxone, was correlated to the treatment period of morphine in rats [24]. The binding of opioids to μ-opioid receptors located on the submucous and myenteric neurons of the intestinal tract induces physiological changes such as impaired gut motility and decreased fluid secretion, leading to OIC [3, 7, 10]. As NAL is a large molecule, its side chains avoid infiltration into the blood-brain barrier, and its bulky structure having a morphinan skeleton selectively inhibits peripheral μ-opioid receptors. It is anticipated that a limited opioid withdrawal response inside the intestinal tract could result from this inhibition by NAL administration. In phase III clinical trials, a significant difference in the clinical opioid withdrawal scale (COWS) was not observed in patients receiving or not receiving NAL [6, 9]. Although COWS is a rating scale evaluating both central and peripheral withdrawal symptoms, it is mainly intended for central withdrawal symptoms. While peripheral withdrawal symptoms were not evaluated in the present study, diarrhea associated with opioid withdrawal syndrome might result from the inhibition of peripheral μ-opioid receptors by NAL.

Clinical guidelines related to OIC, which were published by the American Academy of Pain Medicine, the European Association for Palliative Care, and the American Gastroenterological Association, recommend traditional laxative therapy (osmotic laxatives and stimulant laxatives) as first-line agents [7, 25, 26]. Based on the current evidence, the traditional laxative therapy has an established efficacy as well as safety and cost benefits, and PAMORAs such as NAL are the recommended second-line agents. Therefore, switching to NAL can be considered when the effect of traditional laxative therapy is insufficient.

The present study has some limitations that must be considered. First, this was a retrospective study conducted in a single facility based on medical records. Particularly, since we evaluated the development of diarrhea based on electronic medical records described by physicians and nurses, the possibility of missing information and nonuniformity in evaluator’s skills cannot be ruled out. Evaluations of diarrhea by the patients also inevitably depended on their subjectivity. Second, opioid withdrawal syndromes other than diarrhea have not been evaluated. Finally, it was difficult to exclude the potential effects of other unknown confounders in our retrospective study.

## Conclusions

In conclusion, this study suggested that NAL administration after more than 17 days of opioid therapy is a significant independent risk factor for NAL-induced diarrhea. Therefore, a prolonged duration of opioid therapy before NAL administration could certainly be involved in the increase of NAL-induced diarrhea.

## Supplementary Information

**Additional file 1: Table S1.** Comparison of chemotherapy regimen in the pancreatic cancer patients with and without diarrhea.

## Data Availability

All data generated or analyzed during this study are included in this published article.

## Abbreviations

AEs: Adverse events
AUC: Area under the ROC curve
COWS: Clinical opioid withdrawal scale
CTCAE: Common Terminology Criteria for Adverse Events
GEM: Gemcitabine
IV: Intravenous
NAL: Naldemedine
OIC: Opioid-induced constipation
OR: Odds ratio
PAMORAs: Peripherally acting μ-opioid receptor antagonists
PPI: Proton-pump inhibitor
PS: Performance status
ROC: Receiver operating characteristics
