# Supplementary material for: Opioid therapy duration before naldemedine treatment is a significant independent risk of diarrhea: a retrospective cohort study
Source: J Pharm Health Care Sci. 2021 Feb 1;7:3. doi: 10.1186/s40780-020-00187-3 (PMC7849155; doi:10.1186/s40780-020-00187-3)
Supplement: Supplementary file 1 — Additional file 1: Table S1. Comparison of chemotherapy regimen in the pancreatic cancer patients with and without diarrhea. [file 40780_2020_187_MOESM1_ESM.docx]

**Supplementary Table 1. Chemotherapy regimen in pancreatic cancer patients**

|  | Diarrhea (+) (n=6) | Diarrhea (−) (n=6) | *P* value |
| --- | --- | --- | --- |
| GEM plus S-1 | 2 |  |  |
| S-1 monotherapy | 2 |  |  |
| GEM monotherapy |  | 1 |  |
| nab-PTX plus GEM |  | 1 |  |
| FOLFIRINOX |  | 1 |  |
| Total | 4 (67) | 3 (50) | 1.000 |

Values are presented as number (%).

GEM, gemcitabine; nab-PTX, nanoparticle albumin-bound paclitaxel
